# Items

**Published:** 1887-05

**Authors:** 


### ITEMS.

THE MONUMENT TO DR. BENJAMIN RUSH.

The Committee of the American Medical Association having charge of the project to erect a monument to "America's most distinguished physician," who was also a great statesman and a signer of the Declaration of Independence, is still at work collecting funds for that purpose. Medical-Director A. L. Gihon, of the United States Navy, is chairman of the committee and will report on the progress thus far made, to the Association at its next annual meeting, to be held in Chicago on June 7th. Dr. S. J. Jones, of Chicago, is the representative of the Committee for the State of Illinois, to whom contributions for the monument-fund may be made, at No. 240 Wabash avenue.

ADDRESSES IN THE GENERAL SESSIONS OF THE INTERNATIONAL MEDICAL CONGRESS.

Many of the medical journals in this and other countries have announced the fact that Professor F. Semmola, of Naples, Italy, is to give a general address on "Bacteriology and its Clinical Therapeutics," to the Congress in general session. General addresses are also promised by Dr. Neudorfer, ot Vienna, Austria, on "The Military Medicine of the Present and of the Near Future;" Dr. Esmarch, of Kiel, Germany, on "Bloodless Operations in Surgery;" Dr. Lutaud, of Paris, France, on "The Influence of the Discoveries of American Surgeons on the Development of Gynæcology in Europe,"

and Dr. Austin Flint, of New York, N. Y., on "Fever, Its Cause, Mechanism and Rational Treatment"—all topics of interest to the whole profession.

---

MEDICAL SOCIETIES TO MEET IN CHICAGO:

The Illinois State Medical Society will meet in this city, in the Methodist Church Block, on May 17th, at 10 A. M.

The Association of American Medical Editors will meet in this city on June 6th.

The American Medical Association will hold its annual meeting in Central Music Hall, corner of State and Randolph streets, in Chicago, beginning on June 7th, at 11 o'clock.

The Executive Committee of the Ninth International Medical Congress will meet in Chicago on June 6th.

